# Predictors of short-term mortality after rheumatic heart valve surgery: A single-center retrospective study

**DOI:** 10.1016/j.amsu.2021.01.077

**Published:** 2021-01-26

**Authors:** Khalid S. Ibrahim, Khalid A. Kheirallah, Fadia A. Mayyas, Nizar R. Alwaqfi, Murtada H. Alawami, Qusai M. Aljarrah

**Affiliations:** a Division of Cardiac Surgery, Department of General Surgery and Urology, College of Medicine, Jordan University of Science and Technology and Princess Muna Center for Heart Diseases and Surgery, King Abdullah University Hospital, Jordan; b Department of Public Health and Community Health, College of Medicine, Jordan University of Science and Technology, Jordan; c Department of Clinical Pharmacy, College of Pharmacy, Jordan University of Science and Technology, Jordan; d Prince Sultan Cardiac Center, Riyadh, Saudi Arabia; e Department of Surgery, Faculty of Medicine, Jordan University of Science and Technology, Jordan

**Keywords:** Predictors, Valve surgery, Emergency surgery, Mortality

## Abstract

**Background:**

Valve replacement surgeries holds risks of morbidity and mortality.

**Materials and methods:**

The study cohort included 346 patients who underwent different types of valve surgery, excluding redo and Bentall operations. All operations were performed through a median sternotomy using cardiopulmonary bypass.

**Results:**

Mean patient age was 51.6 ± 16.1 years, and 51% were male. Approximately 21% had diabetes, and 44.6% were hypertensive. Aortic valve replacement (AVR) was performed in 125 patients (37%), mitral valve replacement (MVR) in 95 (28%), combined AVR and MVR in 42 (13%), AVR plus coronary artery bypass grafting (CABG) in 19 (6%), and MVR plus CABG in 32 (10%). Operative mortality was 5.8% (n = 20). In the bivariate-level analysis, older age, operation type, hypertension, emergency surgery, use of a biological valve in the aortic or mitral position, pump time greater than 120 min, and aortic clamp time greater than 60 min were significant predictors of 30-day mortality. Use of medications stratified by duration (less than or more than a month) was also shown to be a predictor of mortality. Use of angiotensin-converting enzyme inhibitors, digoxin, beta-blockers, statins, and loop diuretics was associated with mortality. Older age, emergency/salvage surgery, use of beta-blockers for less than 1 month preoperatively, and use of a biological valve in the aortic position were significant and independent predictors of 30-day mortality.

**Conclusion:**

Age, emergency valve surgery, use of a biological valve, use of beta-blockers for less than 1 month before surgery, type of surgery, EF<35%, pump time, and cross clamp time were all found to be independent predictors of mortality in patients undergoing valve surgery. Further prospective multicenter studies may be needed to provide a comprehensive assessment of mortality in patients undergoing valve surgery in Jordan.

## Highlights

• Rheumatic heart disease is still a health issue in developing countries.
• Developed countries are facing rheumatic patients due to forced immigrations.
• Early diagnosis and early referral to surgery is correlated with better outcomes.
• More than eight predictors were found to be independent predictors of mortality.

## 1 Introduction

Valvular heart disease, both stenosis and regurgitation, can cause a wide range of symptoms, including shortness of breath, chest pain, fatigue, syncopal attacks, and even sudden death. Surgical valve replacement is still the gold-standard treatment for symptomatic valve disease. In Jordan, similar to most developing countries, rheumatic valve disease is still the leading pathology, although the incidence is declining [1].

Several studies have evaluated predictors of mortality after valve replacement surgery [[2], [3], [4]]. In patients undergoing aortic or mitral valve surgery, older age, high creatinine, coronary artery disease and congestive heart failure (HF) were significant independent predictors of in-hospital mortality [3]. We have previously investigated predictors of mortality and morbidity following coronary artery bypass surgery (CABG) in the north of Jordan; these included older age, female sex, HF, prolonged inotropic support, increased left atrial size, and mitral regurgitation [[5], [6], [7]]. Valve surgery is associated with significant mortality; however, predictors have not yet been investigated in the Jordanian population.

In this study, we investigated preoperative, intraoperative, and postoperative predictors of mortality after valve replacement surgery in Jordanian patients.

## 2 Patients and methods

### 2.1 Patients

From 2002 to 2017, 346 patients underwent isolated aortic valve replacement (AVR), isolated mitral valve replacement (MVR), AVR plus CABG, MVR plus CABG, or combined AVR and MVR with or without CABG were included in this cohort retrospective study. The study was conducted at King Abdullah University Hospital (KAUH), located in the north of Jordan. Patients who had previous cardiac surgery and those with aortic dissection requiring aortic root replacement as well as valve repair were excluded, leaving a study cohort of 346 patients for analysis.

Clinical, echocardiographic, and surgical data from the electronic medical records of eligible patients were abstracted and reviewed. Prior use of medications was documented and stratified by duration of less than or more than one month. Short-term mortality was defined as all-cause mortality within 30 days after surgery. Preoperative coronary angiography was performed for patients with an indication and for those older than age 35. The study was approved by the Institute Research Board at KAUH and Jordan University of Science and Technology. The study was registered at the “Research Registry” with unique identification number UIN of 6457. The work has been reported in line with the STROCSS criteria [8].

### 2.2 Operative procedure

All patients had a median sternotomy, cardiopulmonary bypass, and ascending aortic cannulation. A two-stage venous cannula was used in patients undergoing AVR and bicaval cannulation in those undergoing MVR. In patients undergoing valve replacement plus CABG, distal anastomoses to the right coronary artery and circumflex artery were done using 7/0 continuous polypropylene sutures. This was followed by valve replacement and left internal thoracic artery to left anterior descending anastomoses. AVR was performed through a transverse incision in the proximal ascending aorta about 1.5 cm distal to the origin of the right coronary artery, while MVR was performed through an interatrial incision. Pledgeted polyester (2/0) interrupted sutures were used in both the aortic and mitral positions after excision of the valve cusps/leaflets and proper decalcification. Age 65 years was used as the cutoff for choosing a mechanical or biological valve. All surgeries were operated by consultant cardiac surgeons certified by the Jordanian Medical Council, in addition to fellowship training in USA or North Europe and licensed to work as cardiac surgeons in Jordan by the Ministry of Health.

### 2.3 Statistical analysis

Data were analyzed using SPSS version 22. Categorical variables are summarized as frequencies and percentages and continuous variables as mean ± standard deviation. Distribution of independent variables for 30-day mortality is presented using X^2^ or independent sample *t*-test, as appropriate. P-values for the bivariate analyses are reported. Independent variables that were significantly associated with mortality (P < 0.05) were entered in a backward conditional logistic regression model (entry at P = 0.05, removal at P = 0.1). Adjusted odds ratios (AOR) and P-values were reported. Alpha level was set at 0.05 for all analyses.

At the bivariate level, variables that were significantly associated with 30-day mortality were included in the logistic regression model. These included operation type, hypertension, emergency surgery, preoperative and intraoperative aortic balloon-pumping, type of aortic or mitral valve used, pump time, aortic clamp time, re-exploration, and prior use of medications, including aspirin, angiotensin-converting enzyme (ACE) inhibitors, digoxin, beta-blockers, statins, and loop diuretics.

## 3 Theory/ Calculation

There is a rising need to revisit rheumatic heart surgery in developed countries because of the increasing number of refuges immigrations where the disease was still endemic in their native home countries.

Diagnosing these patients early will improve the outcomes from valve replacement surgery. Our study may help in selecting patients who are at increased risk of mortality to provide preventable measures when possible to reduce complications and death.

## 4 Results

### 4.1 Patient characteristics

In this study, mean age was 51.6 ± 16.1 years, and 51% (n = 178) were male. About 21% of patients had diabetes, 44.6% had hypertension (Table 1), 31.6% had coronary artery disease, and 22.5% had HF. AVR was performed in 125 patients (37%), MVR in 95 (28%), combined AVR and MVR in 42 (13%), AVR plus CABG in 19 (6%), and MVR plus CABG in 32 (10%). Operative mortality was 5.8% (n = 20). Cause of death was multi-system organ failure in 10, sepsis in 6, stroke in 1, and undocumented in 3. Most of patients were on standard medical therapy for treatment of comorbidities: aspirin (33.3%), clopidogrel bisulfate (16.2%), statins (25.2%), beta-blockers (35.4), ACE inhibitors (27.8%), and loop diuretics (48.5%).

**Table 1:** Represents demographic data, Pre- Intra- and Postoperative predictors of mortality after rheumatic valvular heart surgery by univariate analysis.

Variables			Not dead		Dead		OR	P-value
		Total	n	%	n	%		
Overall		346	326	94.20%	20	5.80%	–	–
Age in years (mean (SD))		51.56 (16.1)	50.7 (1601)		65.6 (8.8)		1.09	0.000
Gender	Female	168	159	94.6%	9	5.4%	Ref	0.462
	Male	178	167	93.8%	11	6.2%	1.16	
	Total	346	326	94.2%	20	5.8%		
Operation type	AVR	125	120	96.0%	5	4.0%	Ref	0.024
	MVR	95	91	95.8%	4	4.2%	1.05	
	AVR + MVR	42	41	100.0%	1	0.0%	1.12	
	AVR + CABG	19	15	78.9%	4	21.1%	6.40	
	MVR + CABG	32	28	87.5%	4	12.5%	3.43	
	Other	33	21	95.5%	1	4.5%	1.14	
	Total	335	317	94.6%	18	5.4%		
Stable Angina	No	253	243	96.0%	10	4.0%	Ref	0.249
	Yes	76	71	93.4%	5	6.6%	1.71	
	Total	329	314	95.4%	15	4.6%		
Histort of CAD	No	225	213	94.7%	12	5.3%	Ref	0.246
	Yes	104	101	97.1%	3	2.9%	0.53	
	Total	329	314	95.4%	15	4.6%		
Recent MI	No	325	311	95.7%	14	4.3%	Ref	0.311
	Yes	8	7	87.5%	1	12.5%	3.17	
	Total	333	318	95.5%	15	4.5%		
Ever Smoking	No	234	222	94.9%	12	5.1%	Ref	0.339
	Yes	93	90	96.8%	3	3.2%	0.62	
	Total	327	312	95.4%	15	4.6%		
COPD	No	319	304	95.3%	15	4.7%	Ref	0.494
	Yes	15	15	100.0%	0	0.0%	0.95	
	Total	334	319	95.5%	15	4.5%		
Hypertension	No	185	182	98.4%	3	1.6%	Ref	0.005
	Yes	149	137	91.9%	12	8.1%	5.31	
	Total	334	319	95.5%	15	4.5%		
Diabetes	No	259	248	95.8%	11	4.2%	Ref	0.381
	Yes	68	64	94.1%	4	5.9%	1.41	
	Total	327	312	95.4%	15	4.6%		
PVD	No	314	301	95.9%	13	4.1%	Ref	0.114
	Yes	13	11	84.6%	2	15.4%	4.21	
	Total	327	312	95.4%	15	4.6%		
AF	No	273	260	95.2%	13	4.8%	Ref	0.515
	Yes	56	54	96.4%	2	3.6%	0.74	
	Total	329	314	95.4%	15	4.6%		
Pre OP renal impairment	No	306	292	95.4%	14	4.6%	Ref	0.418
	Yes	11	10	90.9%	1	9.1%	2.09	
	Total	317	302	95.3%	15	4.7%		
MR	No	141	133	94.3%	8	5.7%	Ref	0.332
	Grage 1	60	56	93.3%	4	6.7%	1.19	
	Grade 2	55	55	100.0%	0	0.0%	0.00	
	grade 3	40	39	97.5%	1	2.5%	0.43	
	Grade 4	25	23	92.0%	2	8.0%	1.45	
	Total	321	306	95.3%	15	4.7%		
TR	No	219	208	95.0%	11	5.0%	Ref	0.153
	Grage 1	43	40	93.0%	3	7.0%	1.42	
	Grade 2	36	36	100.0%	0	0.0%	0.00	
	grade 3	15	15	100.0%	0	0.0%	0.00	
	Grade 4	4	3	75.0%	1	25.0%	6.30	
	Total	317	302	95.3%	15	4.7%		
AR	no	190	181	95.3%	9	4.7%	Ref	0.126
	Grage 1	55	51	92.7%	4	7.3%	1.58	
	Grade 2	42	40	95.2%	2	4.8%	1.01	
	grade 3	25	25	100.0%	0	0.0%	0.00	
	Grade 4	34	29	85.3%	5	14.7%	3.47	
	Total	346	326	94.2%	20	5.8%		
ER	elective	309	294	95.1%	15	4.9%	Ref	0.041
	urgent	16	13	81.3%	3	18.8%	4.52	
	Total	325	307	94.5%	18	5.5%		
ASA	No	212	206	97.2	6	2.8%	Ref 2.80	
	Yes	106	98	92.5	8	7.5%		
	Total	318	304	95.6	14	4.4%		

Plavix	No	273	263	96.3%	10	3.7%	Ref	0.178
	Discontinued 7 days or less	53	49	92.5%	4	7.5%	2.15	
	Total	326	312	95.7%	14	4.3%		

ACE Inhibitors	No	238	227	95.4%	11	4.6%	Ref	0.027
	less than a month	19	16	84.2%	3	15.8%	3.87	
	More than month	73	72	98.6%	1	1.4%	0.29	
	Total	330	315	95.5%	15	4.5%		
Digoxin	No	211	201	95.3%	10	4.7%	Ref	0.008
	less than a month	15	12	80.0%	3	20.0%	5.03	
	More than month	100	98	98.0%	2	2.0%	0.41	
	Total	326	311	95.4%	15	4.6%		
Aldosterone antagonist	No	269	258	95.9%	11	4.1%	Ref	0.218
	less than a month	5	4	80.0%	1	20.0%	5.86	
	More than month	51	48	94.1%	3	5.9%	1.47	
	Total	325	310	95.4%	15	4.6%		
B Blockers	No	212	207	97.6%	5	2.4%	Ref	0.000
	less than a month	32	26	81.3%	6	18.8%	9.55	
	More than month	84	81	96.4%	3	3.6%	1.53	
	Total	328	314	95.7%	14	4.3%		
Statins	No	246	237	96.3%	9	3.7%	Ref	0.036
	less than a month	28	24	85.7%	4	14.3%	4.39	
	more than month	55	53	96.4%	2	3.6%	0.99	
	Total	329	314	95.4%	15	4.6%		
Loop_Diuretics	No	151	143	94.7%	8	5.3%	Ref	0.003
	less than a month	26	22	84.6%	4	15.4%	3.25	
	more than month	152	150	98.7%	2	1.3%	0.24	
	Total	329	315	95.7%	14	4.3%		
Type valve used Aortic	Mechanical	129	228	97.0%	7	3.0%	Ref	0.001
	Biological	62	98	88.3%	13	11.7%	4.30	
	Total	191	326	94.2%	20	5.8%		
Type valveMitral	Mechanical	120	214	96.4%	8	3.6%	Ref	0.029
	Biological	67	112	90.3%	12	9.7%	2.90	
	Total	187	177	94.2%	20	5.8%		
Pump time	<120min	224	216	96.4%	8	3.6%	Ref	0.030
	>120min	56	53	89.9%	6	10.2%	3.05	
	Total	346	269	95.1%	14	4.9%		
Aorta_Clamp	No	247	237	96.0%	10	1.0%	Ref	0.028
	Yes	99	89	89.9%	10	10.1%	2.67	
	Total	346	326	94.2%	20	5.8%		
Intra Op Transf.	No	258	246	95.3%	12	4.7%	Ref	0.687
	Yes	58	56	96.6%	2	3.4%	0.73	
	Total	316	302	95.6%	14	4.4%		
Reexploration	No	312	299	95.8%	13	4.2%	Ref	0.070
	Yes	10	8	80.0%	2	20.0%	5.75	
	Total	322	307	95.3%	15	4.7%		
Prolonged support	No	266	256	96.2%	10	3.8%	Ref	0.180
	Yes	52	48	92.3%	4	7.7%	2.13	
	Total	318	304	95.6%	14	4.4%		
Post Op Renal Failure	No	311	298	95.8%	13	4.2%	Ref	0.102
	Yes	12	10	83.3%	2	16.7%	4.58	
	Total	323	308	95.4%	15	4.6%		
Pneumonia Sepsis	No	316	303	95.9%	13	4.1%	Ref	0.438
	Yes	13	12	92.3%	1	7.7%	1.94	
	Total	329	315	95.7%	14	4.3%		
Post Op Stroke TIA	No	324	310	95.7%	14	4.3%	Ref	0.171
	Yes	4	3	75.0%	1	25.0%	7.38	
	Total	328	313	95.4%	15	4.6%		
Intra_OP_insertion_of_temporary_pacemaker	No	230	219	95.2%	11	4.8%	Ref	0.764
	Yes	91	88	96.7%	3	3.3%	0.68	
	Total	321	307	95.6%	14	4.4%		
Post_OP_AF	No	278	265	95.3%	13	4.7%	Ref	0.480
	Yes	38	37	97.4%	1	2.6%	0.55	
	Total	316	302	95.6%	14	4.4%		

Data are presented as mean (SD) for continuous variables or percentages for categorical variables. AOR: adjusted odd ratio, AVR: aortic valve repair, MVR: mitral valve repair, CABG: coronary artery bypass surgery, CAD: coronary artery disease, MI: myocardial infarction, COPD: chronic obstructive pulmonary disease, PVD: peripheral vascular disease, AF: atrial fibrillation, MR; mitral regurgitation, TR: tricuspid regurgitation, AR: aortic regurgitation, ER: emergency surgery, Pre op: pre-operative, Post op: post-operative, IABP: intra-aortic balloon pump, TIA: transient ischemic attach, POAF: post-operative atrial fibrillation.

### 4.2 Predictors of mortality

Table 1 presents univariate predictors of mortality 30 days after surgery. At the bivariate level, older age (P < 0.0001), operation type (P = 0.024), hypertension (P = 0.005), emergency/salvage surgery (P = 0.041), use of a biological valve in the aortic (P = 0.001) or mitral (P = 0.029) position, pump time greater than 120 min, and aortic clamp time greater than 60 min were significant predictors of 30-day mortality. Use of medications—ACE inhibitors (P = 0.027), digoxin (P = 0.008), beta-blockers (P < 0.001), statins (P = 0.036), and loop diuretics (P = 0.003)—was also associated with mortality.

We also used logistic regression to identify independent predictors of mortality (Table 2), adjusting for other variables or potential confounders. Older patients (P = 0.028, AOR = 10.6), emergency/salvage surgery (P = 0.034, AOR = 7.12), use of beta-blockers for less than a month before surgery (P = 0.006, AOR = 8.59), and a biological valve in aortic position (P = 0.007, AOR = 7.09) were all significant and independent predictors of 30-day mortality (Table 1).

**Table 2:** Multivariate analysis of risk factors associated with mortality after rheumatic valvular heart surgery.

Patients characteristics	OR	P value
Age, yrs.	1.06	0.028
Emergency surgery	7.12	0.034
Use of β-blockers < one month	8.59	0.006
Use of β-blockers > one month	1.52	0.612
Type of aortic valve Biologic	7.09	0.007
Type of mitral valve Biologic	2.17	0.269
Type of surgery	5.85	0.025
EF<35%	5.21	0.002
Pump time	2.60	0.010
Cross clamp time	3.22	0.006

OR: Odds Ratio, EF: Ejection Fraction.

## 5 Discussion

Valve replacement surgery is the second most commonly performed open heart surgery in Jordan after CABG. However, independent predictors of mortality after valve operations have not been studied as widely as predictors after CABG. This paucity of data might be due to fewer valve operations being performed than CABG procedures as valve operations require more time and/or more centers to accumulate enough cases to draw conclusions. In addition, a wide range of valve procedures are performed, and the risk of mortality may vary with the type of procedure [9]. We have previously evaluated predictors of mortality and morbidity for patients undergoing CABG [[5], [6], [7]]. Predictors of mortality included age >65 years, female sex, HF, left ventricular ejection fraction (LVEF) ≤35%, prolonged inotropic support, mechanical ventilation >2 h, postoperative pneumonia, and postoperative stroke, as well as enlarged left atrial size and mitral regurgitation [[5], [6], [7]]. Previous studies have identified predictors of mortality in different patient populations undergoing valve surgery. In those undergoing mitral valve replacement, postoperative higher creatinine, low cardiac output, small mitral valve size, and new-onset atrial fibrillation were significant independent predictors of morality [10].

Our study, which is the first in Jordan to present results after valve surgery, highlights important preoperative, intraoperative, and postoperative predictors that might increase risk of mortality in patients undergoing various types of valve surgery. Consistent with previous studies [3,4], increased age was found to be a predictor of 30-day mortality in our study population. Older patients have multiple comorbidities with deterioration of organ function. In patients >80 years old, emergency surgery and CABG were the most important predictors of early mortality after mitral valve surgery (both repair and replacement), with estimated mortality of 18% [11]. Similarly, in patients undergoing AVR, emergency surgery, atrial fibrillation, and older age were the strongest predictors of mortality [12]. Most of our cases were rheumatic in nature (89%). This usually starts at a young age, and over time patients develop progressive deterioration in left ventricular function. Left ventricular dysfunction was shown to be a strong predictor of mortality after valve replacement surgery [1]. This might help us understand potential causes of mortality in our relatively young cohort of patients as the pathology starts early in life in contradiction to degenerative valve pathology that starts later in life.

In the present study, patients with preoperative low ejection fraction <35% had higher incidence of mortality that those with normal ejection fraction. These results are replicated in many previous studies [11]. It is obvious that in this relatively young cohort of patients, the low ejection fraction is related to chronic disease process that ended with left ventricular dysfunction. Proper follow up for these patients and early referral to surgery before severe deterioration of the left ventricular function is a key step to better outcomes.

Emergency/salvage surgery was found to be an independent predictor of 30-day mortality, similar to other study results [10, 11, 7]. The pathologies that mandate emergency valve surgery, including acute mitral incompetence following acute myocardial infarction or acute valve incompetence secondary to infective endocarditis, may put the patient in a state of acute HF, increasing the risk of death.

Patients who receive biological valves are usually older than those who receive mechanical valves. However, our model identified both age and biological valves as significant, yet independent, predictors of mortality after adjusting for other variables. In a sub-analysis in which we excluded age from the regression model, the effect of valve type was almost identical to that reported one with age in the model, suggesting that 30-day mortality is affected by age independent of valve type and vice-versa.

Beta-blockers are key medications for treating HF, myocardial infarction, and atrial fibrillation, and are useful adjuncts for hypertension management. Beta-blockers can antagonize the effects of an overactive sympathetic nervous system, which is responsible for development and progression of HF. These medications reduce myocardial oxygen demand and improve LVEF in patients with HF [13] and control heart rate in patients with mitral stenosis [14].

Due to their negative inotropic and chronotropic effects, beta-blockers may initially worsen edema, hypotension, bradycardia, and LVEF before improvement is observed, but subsequent improvement often occurs after 6–12 months of therapy [15]. The incidence and severity of beta-blockers’ adverse effects are usually dose dependent [16]. Thus, patients should be clinically monitored and their dose titrated carefully to avoid adverse outcomes. Given the different effects of beta-blockers, increasing the dosage may cause unintended side effects and significant morbidity, particularly in patients with hypotension and bradycardia, without additional benefits [16].

In our study, most of patients were on high doses of beta blockers. Interestingly, use of beta-blockers for less than 1 month before surgery increased the risk of mortality relative to that of non-users, suggesting that short-term use might worsen symptoms and increase mortality risk. To test this hypothesis, we evaluated the correlation between use of beta-blockers for less than 1 month and LVEF as well as presence of orthopnea and found a positive correlation. Current guidelines recommend beta-blocker therapy for patients with mild to moderate compensated HF, with stable New York Heart Association class II/III symptoms and on standard therapy for HF (diuretics and an ACE inhibitor) [17]. Intriguingly, beta-blockers were found to increase risk of sudden cardiac death and need for surgery in patients with chronic, severe, non-ischemic MR [18].

Interestingly, the type of surgery performed in our patients predicts mortality. The more complex the surgery, the higher the mortality. Whereas the mortality rate was around 2% among patients who underwent isolated AVR, it jumped to 7% in patients with combined AVR, MVR and CABG. These results are replicated in other studies [8]. Prolonged cardiopulmonary bypass time and X-clamp time, which were also found to be independent predictors of mortality, might explain this. In a separate analysis, we removed prolonged bypass time and cross clamp time from the model to avoid confounding, and type of surgery remained a predictor of mortality.

Taylor et al. found postoperative atrial fibrillation (POAF) to be a predictor of mortality after valve replacement surgery [4], whereas Al-Waqfi et al. found no relation [5]. Similarly, we did not find a correlation between POAF and mortality. This might be due to the low prevalence of POAF (12%) in our study.

Our study results help in risk stratification of patients with increased mortality rate and help define how patients’ characteristics, underlying morbidities and type of surgery would affect the clinical outcome of patients undergoing valvular surgery. Meticulous timing for performing valvular surgeries i.e before deterioration of the left ventricular function as well as proper preparation of these patients in terms of adjusting their medications and controlling the modifiable risk factors are key measures toward improving the outcome and reducing mortality. More accurate risk stratification systems are required to identify patients who may benefit from different types of valvular surgeries and or in need of an early treatment.

## 6 Limitations

This is a retrospective study with a limited sample size and some missing data. Data were extracted from a single center, which may not represent short-term mortality in all centers in Jordan.

## 7 Conclusion

Age, emergency valve surgery, use of a biological valve, and use of beta-blockers for less than 1 month before surgery, Type of surgery, EF<35%, Pump time, Cross clamp time were all found to be independent predictors of mortality in patients undergoing valve surgery. Further prospective multicenter studies may be needed to provide a comprehensive assessment of mortality in patients undergoing valve surgery in Jordan. A multicenter prospective studies are needed to investigate these predictors while eliminating the limitations of retrospective studies.

## Declaration of competing interest

NO CONFLICT OF INTEREST OF ANY TYPE FOR ANY OF THE AUTHORS.

## Abbreviations

**AVR**: Aortic Valve Replacement
**MVR**: Mitral Valve Replacement
**CABG**: Coronary Artery Bypass Grafting
**HF**: Heart Failure
**AOR**: Adjusted Odds Ratio
**ACE**: Angiotensin Converting Enzyme
**LVEF**: Left Ventricular Ejection Fraction
**POAF**: Post Operative Atrial Fibrillation

## Registration of research studies

1. Name of the registry: Research Registry
2. Unique Identifying number or registration ID: 6457
3. Hyperlink to your specific registration (must be publicly accessible and will be checked): https://www.researchregistry.com/browse-the-registry#home/
